# *Peganum harmala* L. extract‐based Gold (Au) and Silver (Ag) nanoparticles (NPs): Green synthesis, characterization, and assessment of antibacterial and antifungal properties

**DOI:** 10.1002/fsn3.4112

**Published:** 2024-03-19

**Authors:** Izaz Ullah, Abdur Rauf, Anees Ahmed Khalil, Muhammad Luqman, Md. Rezaul Islam, Hassan A. Hemeg, Zubair Ahmad, Yahya S. Al‐Awthan, Omar Bahattab, Mohammed Mansour Quradha

**Affiliations:** ^1^ Department of Chemistry University of Swabi Anbar Khyber Pakhtunkhwa Pakistan; ^2^ Faculty of Allied Health Sciences, University Institute of Diet and Nutritional Sciences The University of Lahore Lahore Pakistan; ^3^ Department of Agricultural Extension and Rural Studies University of Sargodha Sargodha Pakistan; ^4^ Department of Pharmacy Faculty of Allied Health Sciences, Daffodil International University, Birulia Savar Bangladesh; ^5^ Department of Clinical Laboratory Sciences, College of Applied Medical Sciences Taibah University Al‐Madinah Al‐Monawara Saudi Arabia; ^6^ Department of Biology, Faculty of Science University of Tabuk Tabuk Saudi Arabia; ^7^ College of Education, Seiyun University Seiyun Hadhramawt Yemen; ^8^ Pharmacy Department, Medical Sciences Aljanad University for Science and Technology Taiz Yemen

**Keywords:** antibacterial, antifungal activity, *Peganum harmala*; Ag and Au NPs

## Abstract

During the last decade, nanotechnology has attained a significant place among the scientific community for the biosynthesis of plant‐based nanoparticles owing to its effective, safe, and eco‐friendly nature. Hence, keeping in view the significance of nanotechnology, the current study was conducted to develop, characterize (UV–visible spectroscopy, scanning electron microscopy, Fourier‐transform infrared spectroscopy, and energy‐dispersive X‐ray spectroscopy), and assess the antimicrobial (antibacterial and antifungal) properties of *Peganum harmala* L. Extract‐based Gold (Au) and Silver (Ag) nanoparticles (NPs). Characteristic absorption peaks at 420 and 540 nm revealed the formation of AgNPs and AuNPs, respectively. SEM images revealed that both silver and gold nanoparticles were oval and spherical with average size ranging from 42 to 72 and 12.6 to 35.7 nm, respectively. Similarly, FT‐IR spectra revealed that the functional groups such as hydroxyl, carboxyl, and polyphenolic groups of biomolecules present in the extract are possibly responsible for reducing metallic ions and the formation of nanoparticles. Likewise, the EDX analysis confirmed the presence of silver and gold in synthesized NPs. Furthermore, the AgNPs and AuNPs showed good antibacterial and antifungal activities. The maximum antibacterial and antifungal activity was noticed for *P. harmala* extract against *Pseudomonas aeroginosa* (21 mm) and *Candida albicon* (18 mm), respectively. Whereas, the maximum antibacterial and antifungal activities of synthesized AgNPs were observed against *Salmonella typhi* (25 mm) and *Penicillium notatum* (36 mm), respectively. Moreover, in the case of AuNPs, the highest antibacterial and antifungal activity of synthesized AuNPs was noticed against *Escherichia coli* (25 mm) and *C. albicon* (31 mm), respectively. Findings of this study revealed that *P. harmala* extract and biosynthesized NPs (silver and gold) possessed significant antibacterial and antifungal properties against different bacterial (*Bacillus subtilis*, *Staphylococcus aureus*, *E. coli*, *P. aeroginosa*, and *S. typhi*) and fungal (*C. albicans*, *Aspergillus Niger*, and *P. notatum*) strains. Further studies must be carried out to assess the probable mechanism of action associated with these antimicrobial properties.

## Graphical abstract

This manuscript highlights the synthesis of *Peganum harmala* L. Extract‐based Gold (Au) and Silver (Ag) nanoparticles (NPs). These nanoparticles were characterized using UV–visible spectroscopy, scanning electron microscopy, Fourier‐transform infrared spectroscopy, and energy‐dispersive X‐ray spectroscopy and were later evaluated for their antimicrobial properties. Results revealed that both synthesized nanoparticles possessed significant antibacterial and antifungal properties.

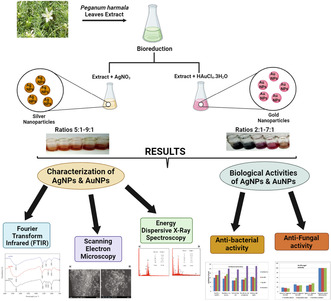

## 1 INTRODUCTION

During the last two decades, nanotechnology has played a vital role in the scientific community for the development of novel drugs. Literature reveals that advanced attributes of the materials can be achieved when operating at nano‐scale owing to the associated quantum size of materials. In modern scientific era, nanotechnology is playing a significant part in diverse fields including biological sciences, physical sciences, and engineering (Ahmad, Ali, et al., 2010; Ahmad, Waheed, et al., 2010). Nanotechnology has signified opportunities for advancements in areas like synthetic materials and biological systems (Ansari et al., 2012). Recently, nanotechnology has been employed for the development of biosensors and biomedicines to overcome the challenges faced during drug delivery and management of different abnormalities. Moreover, nanotechnology has also imparted significant impact in the field of photo devices, physics, electronics, and energy materials (Asharani et al., 2008). In recent times, various medicinal plants have been used for the synthesis of nanoparticles owing to the emergence of green chemistry. For this purpose, extracts of different parts of several plants have been used for the biosynthesis of metallic nanoparticles (Alomar et al., 2020). Diverse noble metals such as silver, gold, palladium, and platinum have extensively been used for the biosynthesis of plant‐based nanoparticles (Zhang et al., 2016).

Generally metallic nanoparticles offer unique properties that make them attractive for various applications, their potential toxicity issues are significant considerations. Therefore, the need to synthesize safe and co‐friendly metallic nanoparticles is considered a top priority of the scientific community. Purposely, the biosynthesis of silver nanoparticles has attained a hallmark position for scientists owing to their safe and environment‐friendly synthesis (Murugan & Shanmugasundaram, 2014; Zangeneh et al., 2019). In medical science, metallic nanoparticles are used for their catalytic degradation, anticancer, antibacterial, antiviral, and anticancer properties. Researchers are focusing on the development of metallic nanoparticles due to their reduced particle size, enhanced surface area, and improved chemical and physical properties. Silver and gold nanoparticles are being used industrially for the production of biocatalysts and biomedicines. Moreover, they are employed in food packaging and as antimicrobial agents in medical devices (Alhumaydhi et al., 2021; Bawazeer et al., 2022).

Since ancient times, various plants have been used by folk people for the treatment of different ailments like constipation, inflammation, diabetes, and microbial infections (Alomar et al., 2020; Hagh‐Nazari et al., 2017; Zangeneh et al., 2018). Plant‐based silver (AgNPs) and gold (AuNPs) nanoparticles are among the easiest and most frequently prepared in research and development (Hano & Abbasi, 2021; Singh et al., 2021). Plant extracts and isolates are considered to be viable alternatives to synthetic drugs owing to their safe nature (Goorani et al., 2019; Zangeneh et al., 2018). Various plant extracts such as *Ulva flexuosa*, *Withania somniferous*, *Calendula officinalis*, *Melia azedarach*, *Euphorbia antiquorum*, and *Silybum marianum* have been used for the synthesis of metallic nanoparticles (Prabhu & Poulose, 2012). In comparison to conventional procedure, green biosynthesis is considered to be eco‐friendly, cost‐efficient, rapid, and safe for the formulation of nanoparticles (Jha et al., 2009). Plant extracts are characterized as safe, eco‐friendly, and economical alternatives to other biological sources such as fungi, algae, and bacteria (Ahmad, Ali, et al., 2010; Ahmad, Waheed, et al., 2010).

*Peganum harmala*, a glabrous plant, is frequently cultivated throughout the eastern Mediterranean region. *P. harmala* is extensively distributed in semi‐soiled areas and belongs to the family *Zygophyllaceae*. In various central Asian and North African regions, *P. harmala* has been prescribed by indigenous practitioners as a valuable medicinal plant since ancient times (Hemmati et al., 2020). This plant has been administrated for the treatment of hypertension and other heart‐related diseases. Various studies have shown the potential of *P. harmala* extract as an effective agent in lowering hypertension and possessing antigenic properties (Yusof et al., 2010). Accordingly, Nayem et al. (2020) showed that the extract of this plant possesses the potential in managing elevated blood pressure and aid vascular resistivity (Nayem et al., 2020). The utilization of *P. harmala* extract as a platform for nanoparticle synthesis holds significant promise due to its unique chemical composition and bioactive properties. *P. harmala* has garnered attention in the field of green synthesis for its ability to facilitate the reduction and stabilization of metallic nanoparticles, offering advantages over plants because of cost effectiveness, easy availability, and versatile phytochemical profile. In this study, we aimed to harness the therapeutic potential of *P. harmala* extract for the green synthesis of Ag and Au nanoparticles, and subsequently evaluate their antimicrobial activities. The novelty of our study lies in the comprehensive exploration of *P. harmala* extract‐mediated green synthesis of Ag and Au NPs and their subsequent assessment for antimicrobial properties. While previous research has highlighted the potential of various plant extracts for nanoparticle synthesis, our study uniquely focuses on *P. harmala*, a plant known for its medicinal properties, particularly in cardiovascular health (Nayem et al., 2020). By leveraging the bioactive constituents of *P. harmala* extract, we successfully synthesized Ag and Au nanoparticles using a green and sustainable approach. Our study offers a systematic characterization of the synthesized nanoparticles using advanced analytical techniques, including scanning electron microscopy (SEM), Fourier‐transform infrared spectroscopy (FT‐IR), UV–visible spectroscopy, and energy‐dispersive X‐ray spectroscopy (EDX). Furthermore, the evaluation of the antimicrobial activities of the synthesized nanoparticles against a diverse panel of bacterial and fungal strains adds significant value to the existing literature. Overall, our study contributes to the growing body of knowledge on green synthesis methodologies and underscores the potential of *P. harmala* extract‐mediated nanoparticle synthesis for biomedical applications, particularly in antimicrobial therapy.

## 2 EXPERIMENTAL

### 2.1 Chemicals and reagents

All the chemicals and reagents used in this study were analytical grade. Methanol and Hydrogen tetrachloroaurate trihydrate [HAuCl_4_·3H2O] were purchased from Merck, Germany. While, Sodium chloride, lead chloride, manganese chloride, and potassium chloride were procured from Sigma, Germany.

### 2.2 Collection, identification, and extraction

In October 2020, a mature *P. harmala* plant was gathered from Swabi, KPK, Pakistan. The plant was identified by Mr. Muhammad Irfan, a lecturer in the Department of Botany, University of Swabi, KPK, Pakistan. The voucher specimen number UOS/Bot‐2111 was deposited in the herbarium of said Department. Leaves (1 kg) were shade‐dried and turned to powder using pestle and mortar. Afterward, powder (4 g) was steeped in distilled water (400 mL) for 2 weeks. The mixture was filtered using Whatman filter paper to separate the soluble part from the insoluble portion. The filtration procedure was repeated three times for effective collection of extract. Later, the collected extracts were used for further analysis and biosynthesis of nanoparticles.

### 2.3 Phytochemical analysis

The resultant extract was evaluated for the presence of secondary constituents through procedures adopted by Adebayo et al. (2009).

#### 2.3.1 Alkaloids

0.5 g of *P. harmala* plant extract was heated in the presence of H_2_SO_4_ (2%) followed by mixing with Dragendorff's reagent. The appearance of orange‐red precipitates showed the presence of alkaloids.

#### 2.3.2 Tannins

0.2 g of *P. harmala* plant extract was boiled and filtered. Ferric chloride was added dropwise in each filtrate. The presence of tannins was indicated through the appearance of a dark green color.

#### 2.3.3 Anthraquinones

0.5 g of *P. harmala* plant extract was heated in HCl (10%) for a short duration. The heated solution was cooled followed by filtration and the addition of chloroform. Ammonia (10%) was mixed with each mixture and heated again. The appearance of pink color revealed the presence of anthraquinones.

#### 2.3.4 Glycosides

Each extract was hydrolyzed using HCl and further neutralized with NaOH solution. Fehling's solution (A: 1 mL, B: 2 mL) was added in small amounts to each extract (2 mL) and boiled. The appearance of crimson precipitates revealed the presence of glycosides.

#### 2.3.5 Saponins

0.5 g of *P. harmala*'*s* extract was mixed with distilled water followed by boiling. Foaming revealed the presence of saponins in each extract.

#### 2.3.6 Test for carbohydrates

0.5 g of *P. harmala*'*s* extract was boiled in a water bath with Fehling's solutions A and B. Formation of brick red color revealed the presence of carbohydrates.

#### 2.3.7 Test for soluble starch

5% KOH was added to a small amount of each extract before being heated, cooled, and acidified with H_2_SO_4_. It was assumed that a yellow hue indicated the existence of soluble starch.

#### 2.3.8 Flavonoids

About 0.2 g of crude extracts were dissolved in dilute NaOH with a few drops of HCl. Flavonoids were found in both extracts as evidenced by the observation of a yellowish solution that became colorless when HCl was added.

#### 2.3.9 Phlobatanins

About 0.5 g of crude extracts were diluted in distilled water and then filtered. With HCl (2%) solution, the filtrates were heated until they boiled. No red precipitate crystallized, which was a sign that phlorotannins weren't present.

#### 2.3.10 Steroids

0.5 g of plant extracts were added together with 2 mL of H_2_SO_4_ and acetic anhydride. The presence of steroids was established by the creation of blue color from violet.

#### 2.3.11 Terpenoids

To create a layer, 0.2 g of crude plant extracts, 2 mL of chloroform, and 3 mL of pure H_2_SO_4_ were carefully combined. The interface developed a reddish‐brown hue, indicating the presence of terpenoids.

#### 2.3.12 Test for coumarin

Each extract's (2 mL aqueous solution) volume was increased by 3 mL of 10% NaOH. Yellow color development indicated the presence of coumarin.

#### 2.3.13 Test for emodin

After combining 0.5 g of the crude extracts with 2 mL of NH_4_OH, 3 mL of benzene was added. The appearance of red color indicated the existence of emodin.

#### 2.3.14 Test for anthocyanin and betacyanin

Using a gas hob, 0.2 g of plant extracts and 1 mL of two regular solutions (2 N) NaOH were combined and cooked for 5 min at 100°C. Anthocyanins were found to be present because of the production of a bluish‐green color, while betacyanins were shown to be present because of the formation of a yellow color.

#### 2.3.15 Reducing sugars

Filtering was done after shaking crude extracts with distilled water. The filtrates were mixed with a few drops of Fehling's solutions A and B before the mixtures were heated for a short period of time. The presence of reducing sugars was demonstrated by the presence of orange‐red precipitates.

### 2.4 Green synthesis of NPs

#### 2.4.1 Green synthesis of AgNPs using aqueous extract of *P. harmala*

2 mg of extract was dissolved in 100 mL distilled water to prepare stock solution. We used distilled water to make 1 mM AgNO_3_ solution. Silver and extract solution were mixed in various ratios to make different fractions of the preparations such as 5:1, 6:1, 7:1, 8:1, and 9:1. The fraction was made in vials and stirred. The production of nanoparticles is indicated by a change in color in each vial's reaction mixture. For the confirmation of silver nanoparticle synthesis, the solutions were tested with a UV–visible spectrophotometer. The best preparation of AgNPs was found at a 7:1 ratio according to the UV–visible spectrophotometer results. Characterization and other analyses of the produced silver nanoparticles were also performed.

#### 2.4.2 Green synthesis of AuNPs from aqueous extract of *P. harmala*

In the descriptive experiment, 1 mM aqueous chloroauric acid (HAuCl_4_) solution was added to the stock aqueous solution of leaves extract in different ratios of 2:1, 3:1, 4:1, 6:1, 7:1 and stirred/stimulated on magnetic stirrer continuously for 12 h. The leaf extract reduced the gold ions into gold nanoparticles. The nanoparticle formation was visually confirmed by the modification in color and by measuring with UV–A visible spectrophotometer in the wavelength range 450–800 nm for gold nanoparticles. The optimal AuNP preparation was found to be 4:1. Instruments like the FTIR, SEM, and EDX were used to characterize AuNPs.

### 2.5 Optimization

The synthesis of silver and gold nanoparticles was examined under different temperatures, salts, pH, and different concentrations of salt (NaCl) to determine the optimum values of all these. Additionally, spectrophotometric measurements were made of the effects of various salts, NaCl, temperature, and pH on the synthesized NPs. The impact was assessed using UV–visible spectra after the various molar solutions of NaCl and other salts were produced. By maintaining the plant ratio constant and vice versa, the impact of an increase in the ratio of gold and silver NPs colloidal was also assessed. At each of the three pH levels—acidic, basic, and neutral—the impact of the pH shift was also assessed. Utilizing NPs at various temperatures allowed researchers to evaluate the impact of temperature.

### 2.6 Characterization of Ag and Au NPs

*P. harmala* extracts were introduced for 2–12 h at a specific temperature in a solution containing Ag and Au salts. The change in color indicated the synthesis of nanoparticles. The Ag and Au nanoparticles generated by green synthesis were characterized using several characterization techniques. The UV–visible spectroscopy approach was used to validate the nanoparticle production technique; the FTIR technique was utilized to assess group stability, and the EDX technique was used to assess composition. SEM is used to measure the size.

#### 2.6.1 UV–Visible spectroscopy

To examine nanoparticles, a wavelength range of 200–800 nm was utilized. By developing a ruby‐red hue, AgNPs were visually verified to have formed. To verify the creation of the AuNPs, the absorbance was scanned at wavelengths between 200 and 800 nm. Quartz cuvettes with an optical path 1 cm long were used for the spectroscopic studies of the silver and gold nanoparticles.

#### 2.6.2 Scanning electron microscopy (SEM)

Using an S‐4800 JEOL scanning electron microscope from the University of Peshawar, the sizes and morphological surfaces of nanoparticles were measured.

#### 2.6.3 Fourier transform infrared (FTIR) spectrometric

Using a Shimadzu FTIR – 8400‐S (Bacha Khan University Charsadda) Fourier transform spectrometer, the detection of functional groups present in green synthesized AuNPs and AgNPs was assessed. Two optically circular flat plates were used to hold the prepared samples. The signals picked up by the computer revealed the outcomes. The frequency range was between 500 and 4000 cm^−1^.

#### 2.6.4 Energy‐dispersive X‐ray (EDX)

The geometry and morphology of Au and AgNPs were also determined. The preparation and identification of the colloids of Au and AgNPs in energy‐dispersive X‐ray spectroscopy was done using a Bruker X‐flash. The electron beams 15 keV energy was maintained for EDX and imaging.

### 2.7 Biological assays

Used the disc diffusion method to determine the pharmacological of the extract and synthesized nanoparticles. These activities were conducted in the Department of Microbiology, University of Hazara.

#### 2.7.1 Antibacterial activity

The zone of inhibition of AgNPs and AuNPs was determined in the microbiological activity. *Staphylococcus aureus* and *Escherichia coli* were the bacteria used in the microbiological experiment. Micro dilutions were used to create the NPs. Nutrient agar was used to create the bacterial culture, which was then incubated for 24 h at 37°C. The NP‐containing petri plates were similarly incubated for 24 h at 37°C. AuNPs and AgNPs had their MBC measured. The region of inhibition was tested using the lowest doses or dilutions of Au and AgNPs. To acquire accurate findings, the procedure was performed three times.

#### 2.7.2 Antifungal activity

The fungicidal activity was determined using the microdilution plate test technique. In the test, *Candida albicans* was employed as the fungus. A 20 mM sodium phosphate buffer solution was combined with 20 L of gold and silver nanoparticle dilutions of 5, 2.5, and 1.25 mg/mL in water. The sample was incubated for 2 h at 37°C.

## 3 RESULTS AND DISCUSSION

### 3.1 Phytochemical screening

For phytochemical screening, the crude extract and different fractions were evaluated. The MeOH extract indicated the presence of tannins, anthraquinones, alkaloids, saponins, anthocyanins, phlobatanins, steroids, coumarins, betacyanins, proteins, and combined reducing sugars. In n‐hexane extract, the presence of reducing sugar, steroids, and terpenoids were indicated. The aqueous extract showed the presence of saponins, terpenoids, combined reducing sugars alkaloids, emodins, anthraquinones, phlorotannins, and coumarins. Normally the polar secondary metabolites were found in all polar extracts. The phytochemical profile of the selected plants is represented in Table 1.

**TABLE 1:** Phytochemical screening of *Peganum harmala* crude extracts.

Phytochemicals	*n*‐hexane	MeOH	H_2_O
Alkaloids	−	+	+
Tannins	−	+	−
Anthraquinones	−	+	+
Glycosides	−	−	−
Reducing sugars	+	−	−
Saponins	−	+	+
Flavonoids	−	−	−
Phlobatanins	−	+	+
Steroids	+	+	−
Terpenoids	+	+	+
Coumarin	−	−	+
Emodin	−	−	+
Anthocyanins and betacyanin	−	+	−
Carbohydrates	−	+	+
Soluble starch	−	−	−
Betacyanin	−	−	−

### 3.2 Synthesis of silver and gold nanoparticles Using *P. Harmala* leaves extracts

**FIGURE 1:**
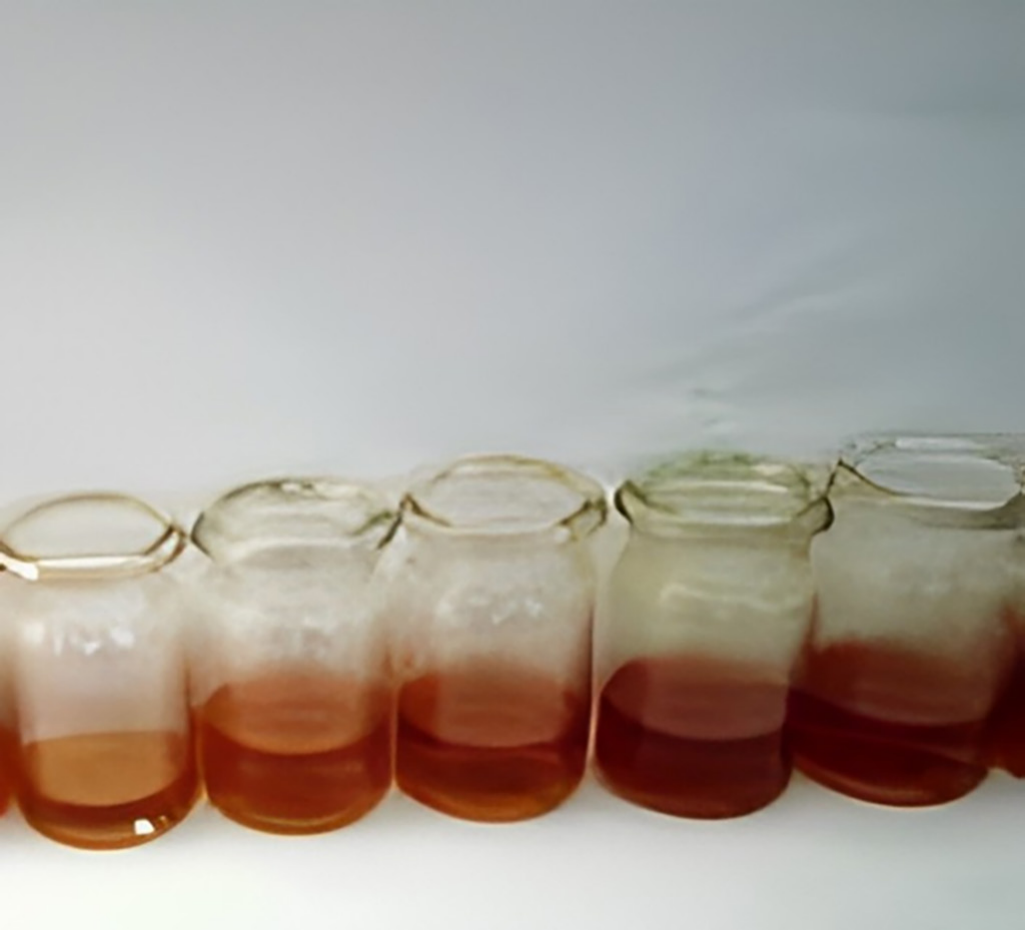
Observation of color transformation from clear to dark.

In the current study, *P. harmala* leaves extract was taken for the reduction purpose of AgNO_3_ to synthesize silver nanoparticles. For the synthesis of Au nanoparticles, the *P. harmala* extract was mixed with a solution of HAuCl_4_·3H_2_O and incubated with different salt solutions. For this determination, aqueous extract solution was used and mixed for 24 h. After mixing the solutions, the silver nitrate solution color is changed to brownish color as shown in Figure 1.

### 3.3 Optimization of various parameters for synthesis of silver nanoparticles

Initially, numerous factors were used to optimize the silver nanoparticle synthesis. During the experiments, the solution of AgNO_3_ and leaves extract were reacted in various ratios (5:1, 6:1, 7:1, 8:1, and 9:1). Gradual changes in color from yellow to reddish yellow and from reddish yellow to dark brown were observed for aqueous solution mixture of AgNO_3_ and leaves extract of the plant. The whole conversion of the aqueous solution from yellowish brown to blackish brown designates the complete formation of silver nanoparticles (AgNPs).

#### 3.3.1 UV–Visible spectroscopy of Ag NPs

The full bio‐reduction of silver ions to silver nanoparticles in samples from the various mixtures of silver nitrate solution and plant extract was observed. By scanning a UV–visible spectrophotometer, silver nanoparticles were initially characterized.

**FIGURE 2:**
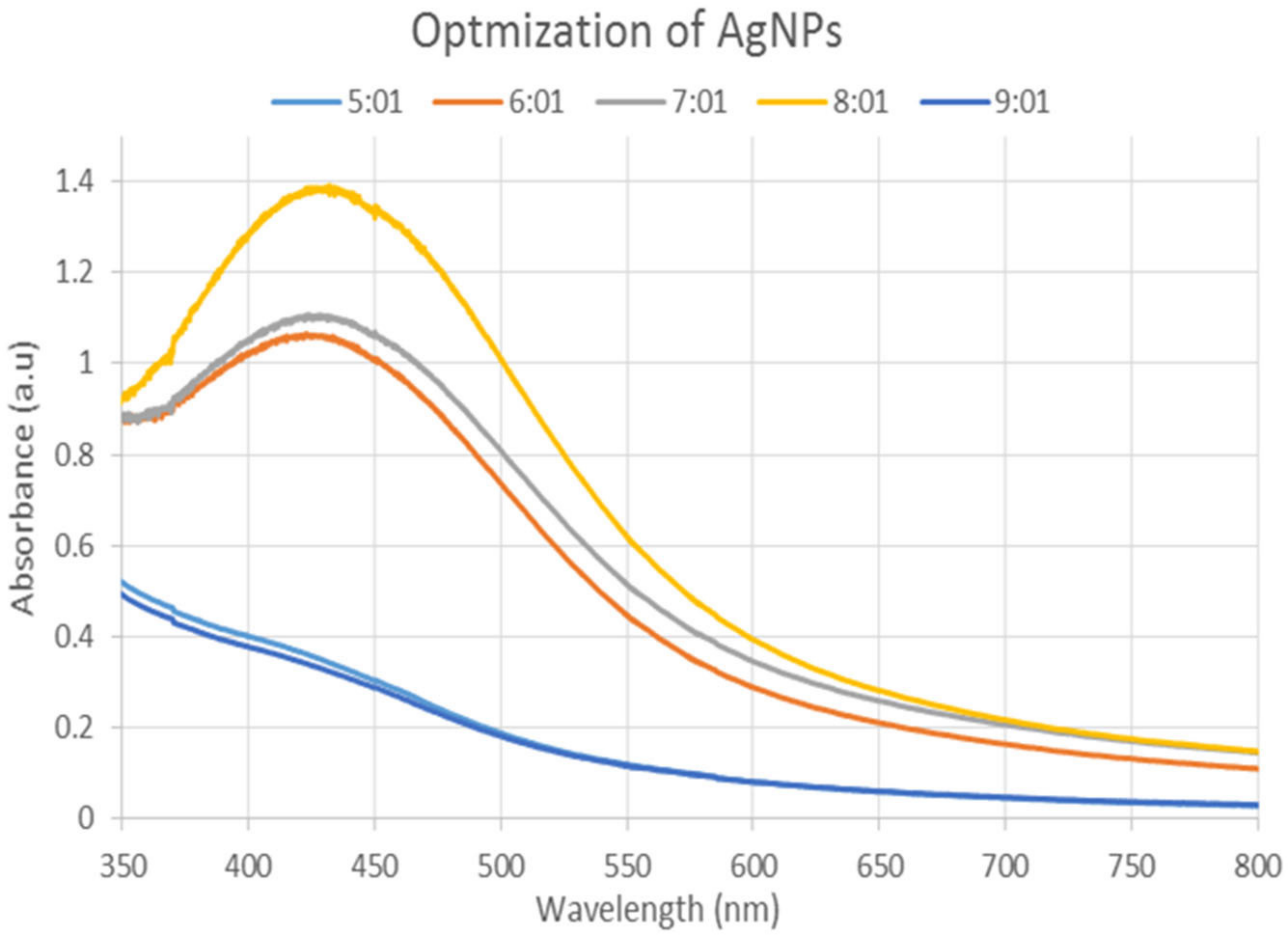
UV–Visible spectrum of silver nanoparticles synthesized by mixing leaves extract and AgNO_3_ solutions in different ratios.

The varying ratios of the reaction mixture (1:1 to 9:1) displayed various strengths of the absorption bands, demonstrating that the plant extract was used to create the silver nanoparticles (Figure 2). The highest surface plasmon absorption band, which is in the absorbance range (400–500 nm) for silver nanoparticles, was provided by an 8:1 ratio at a maximum of 425 nm. The instantaneous transformation of the substance's color from creamy yellow to brownish, which over time becomes entirely dark reddish brown, shows that the creation of silver nanoparticles began. According to UV–visible spectrophotometry, the reaction mixture's brownish color of silver nanoparticles is caused by the stimulation of surface plasmon resonance.

#### 3.3.2 Effect of salt concentration on silver nanoparticles

**FIGURE 3:**
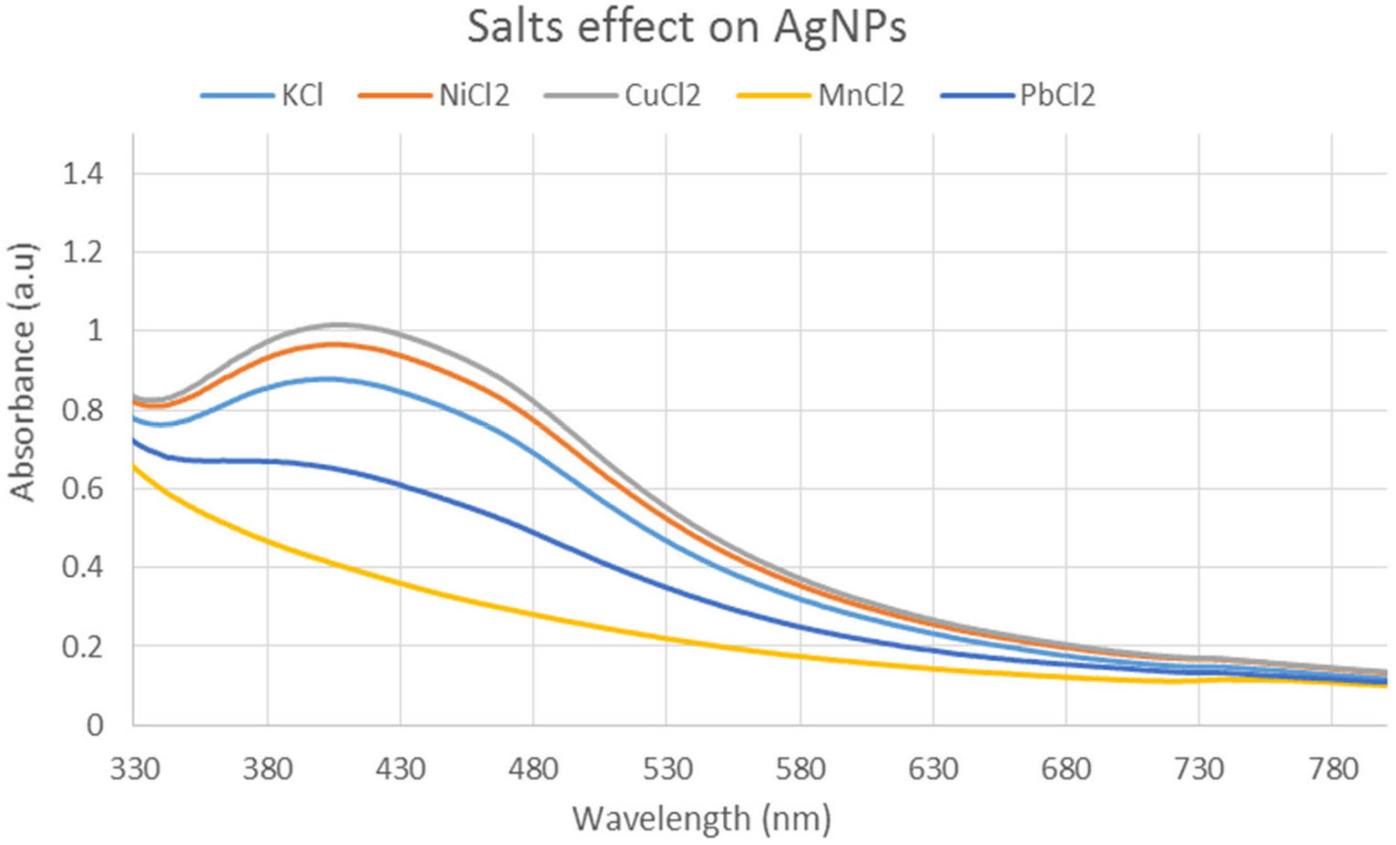
UV–Visible spectrum showing the effect of different salts on silver nanoparticles.

The impact of varying ionic strengths on the size of silver NPs was also investigated. Because the important environmental circumstances may change depending on the salts and their ionic strength, examination of NPs in different salts ranges of ionic strength is also required for assessment of NP behavior in diverse salt environments. The salts used in the evaluation of the effects on silver NPs are NiCl_2_, CuCl_2_, KCl, MnCl_2_, and PbCl_2_. The differences in the particle size as a function of the ionic strength were shown in spectra (Figure 3).

#### 3.3.3 Heat effect on silver nanoparticles

**FIGURE 4:**
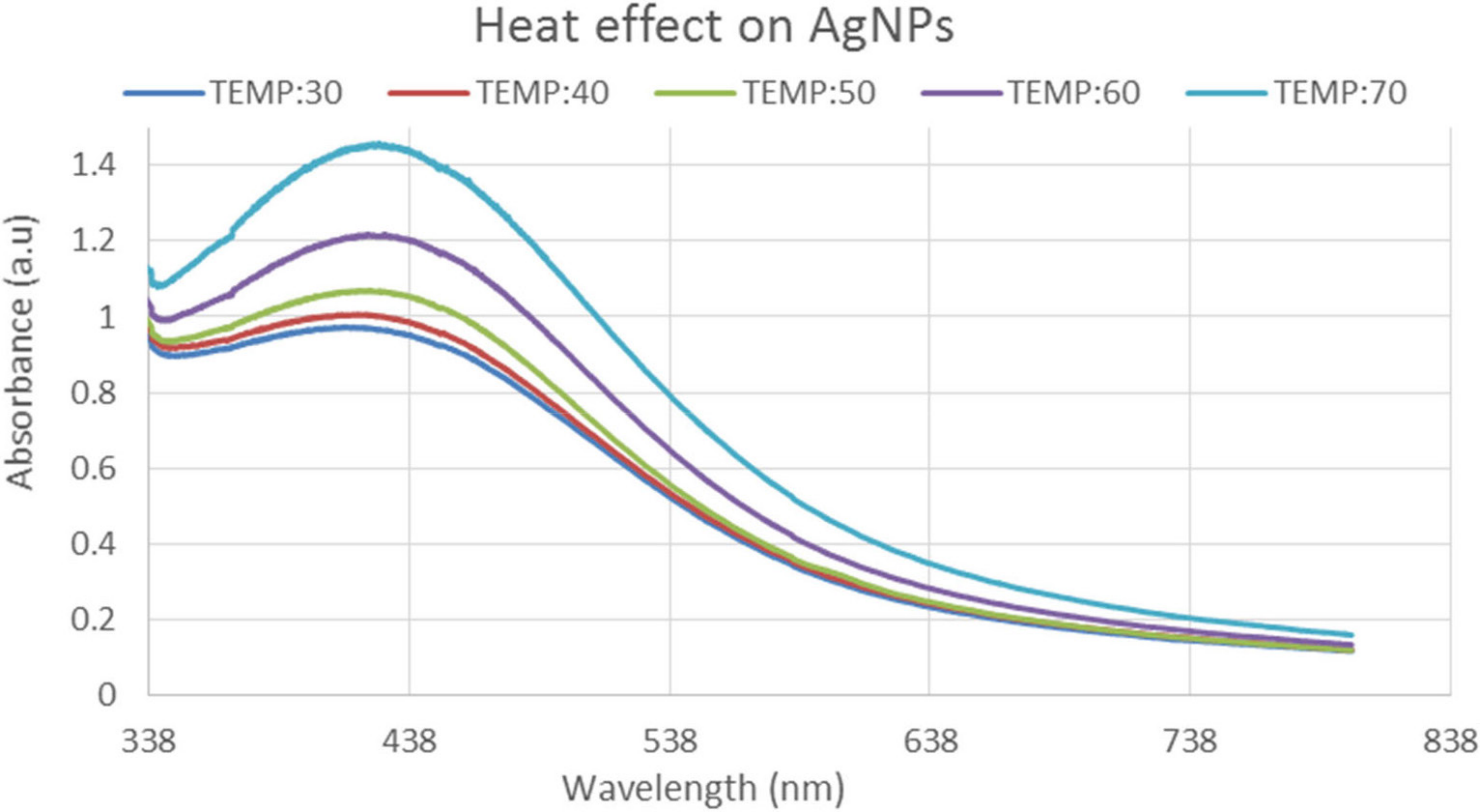
UV–Visible spectrum for effect of heat on Ag nanoparticle solution.

The effects of heat on the silver nanoparticles are then investigated at various temperatures. The suspension of AgNPs solution was exposed to various temperatures (30, 40, 50, 60, and 70°C). The silver nanoparticles are unstable at high temperatures such as 80 and 90°C, but they become more stable as the temperature drops below 70°C. Only at two variants of temperature i.e.; 60 and 70°C, produce maximum absorbance peaks of 1.2 and 1.4 a.u., at 450 nm wavelengths respectively, as shown by UV–visible spectroscopy data. The creation of large‐sized NPs was indicated by a broadening of the spectrum peak, and the generation of small‐sized NPs by a narrowing of the spectrum. At higher temperatures, the rate of reduction of silver ions was higher. The highest peaks are observed at 70°C, while the lowest peaks are observed at 30 and 40°C, respectively, as indicated in Figure 4.

#### 3.3.4 pH effect of silver nanoparticles

**FIGURE 5:**
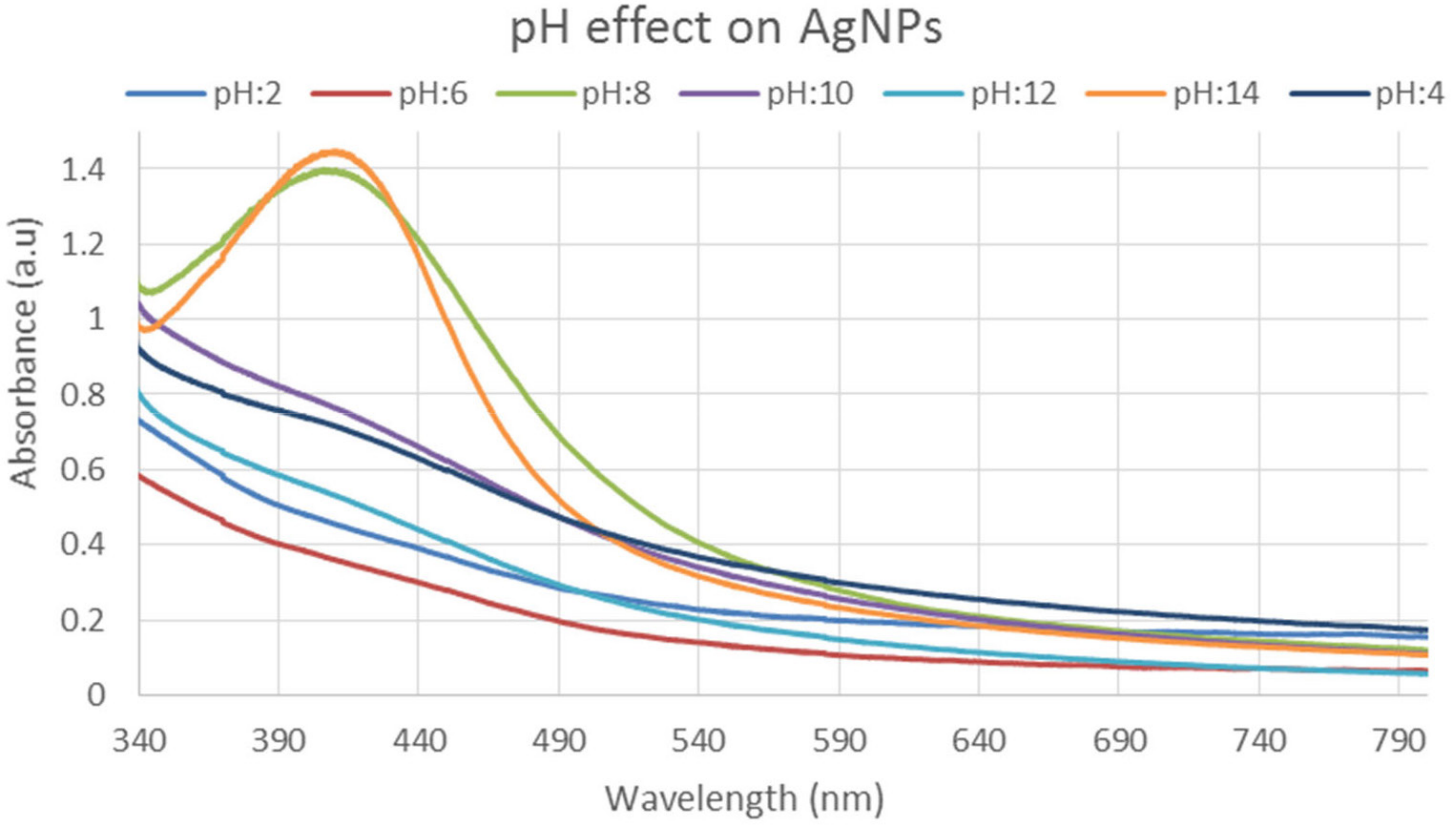
UV–Visible spectrum showing the effect of different pH on silver nanoparticles.

UV–visible spectroscopy was used to study the influence of synthesized silver nanoparticles in various pH solutions. As illustrated in Figure 5, several pH solutions with values of 2, 4, 6, 8, 10, 12, and 14 were placed in beakers from left to right. The greatest peak was detected at 440 nm wavelengths for a pure sample with an absorbance of 18.8 a.u., according to UV–visible spectroscopy measurements. After a pure sample at 430 nm wavelength with an absorbance value of 1.45 a.u., the highest peaks are detected at pH 8 and pH 14. In a spectrum, the solution with pH 8 and 14 had more steady peaks, indicating excellent nanoparticles. The AgNPs were stable at basic conditions. However, the absorption reduced when the pH dropped below 6.0. At higher pHs, we also noticed narrow peaks and strong absorption. All reaming pH solutions, with the exception of these two, showed a decrease in absorbance in the 350–700 nm regions as shown in the inset of Figure 5.

#### 3.3.5 Effect of NaCl on silver nanoparticles

**FIGURE 6:**
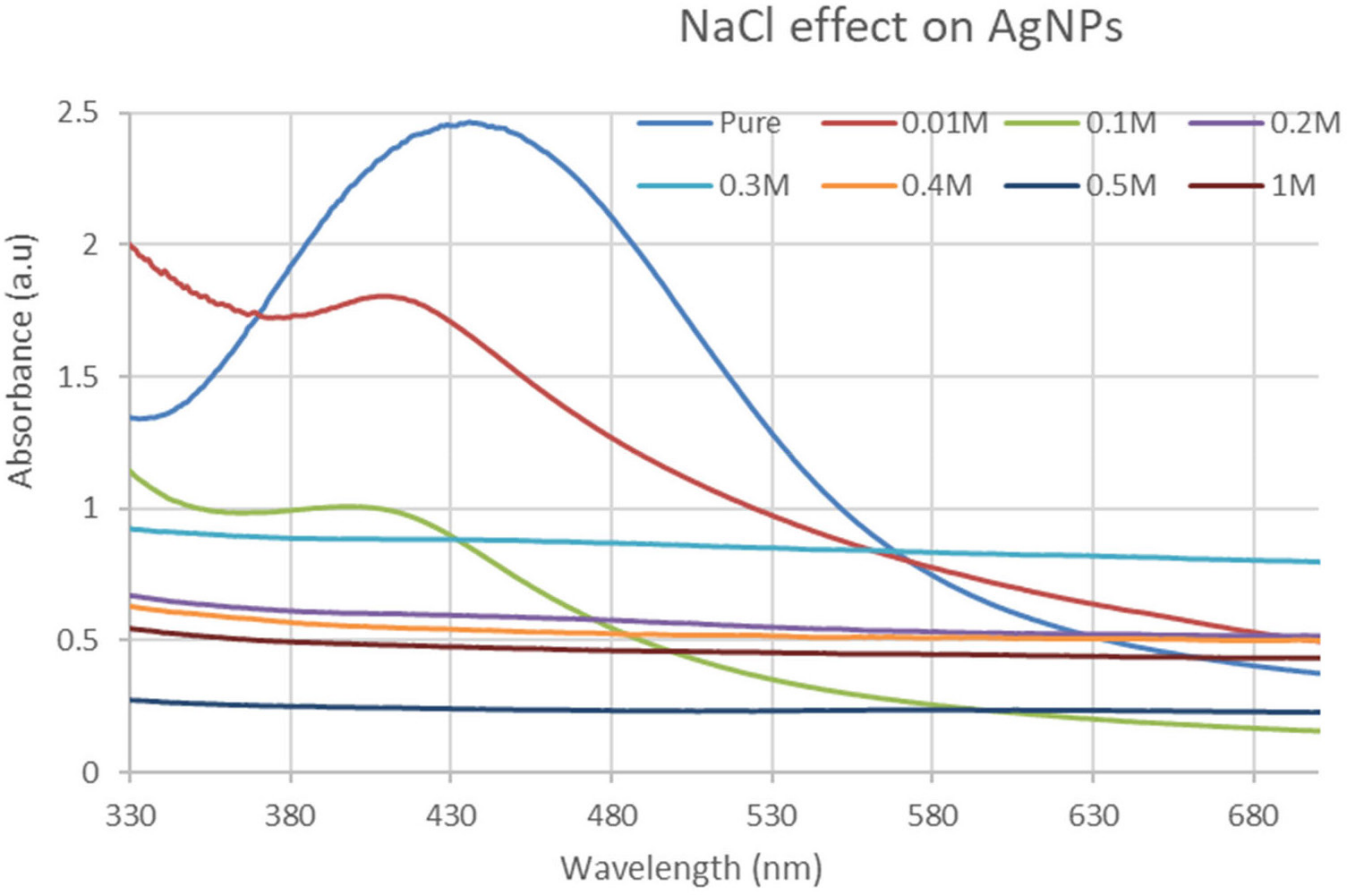
UV–Visible spectrum showing the effect of different concentrations of NaCl solution on silver nanoparticles.

The silver nanoparticle solution was combined with NaCl at varied ratios of 0.01, 0.1, 0.2, 0.3, 0.4, 0.5, and 1.0 to see what effect it had. As the concentration ratio rises, the color shifts from left to right. The spectra of these solutions were obtained using UV–visible spectroscopy. When the salt concentration was increased, the absorbance value at 420 nm wavelength drops, according to the UV–visible spectrum. The 0.01 concentration solution, as it can be concluded, has a higher absorbance value than the other concentrations. At 0.5 and 1.0 concentrations, the absorbance values are nearly identical. However, the graph shows that the NaCl treatment reduces the intensity of the peaks as shown in Figure 6.

### 3.4 Optimization of gold nanoparticles

**FIGURE 7:**
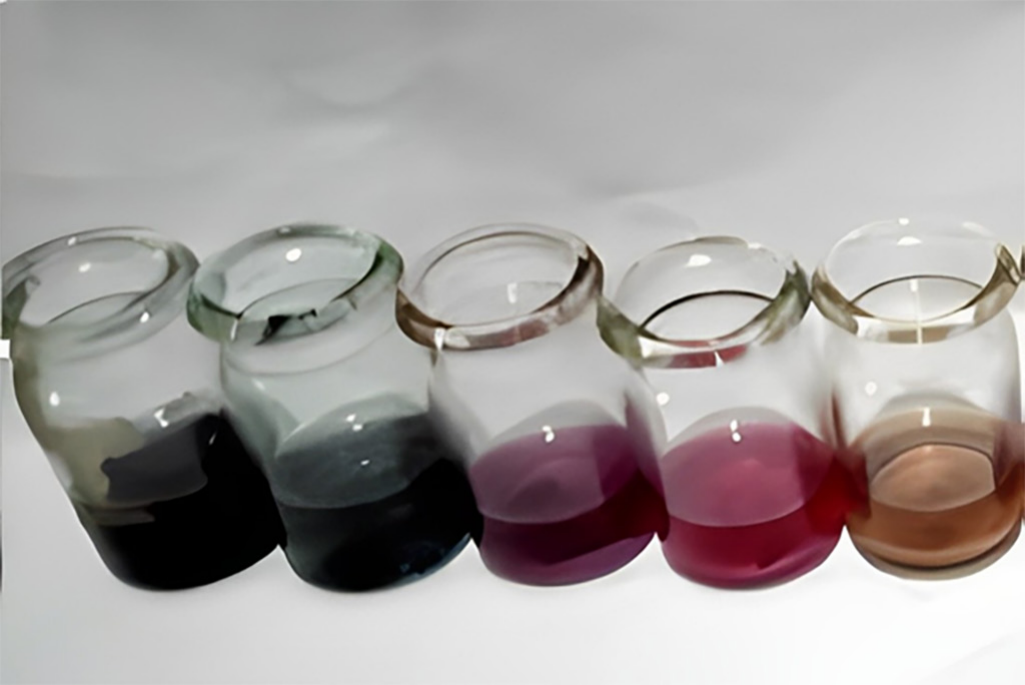
Optimization of gold nanoparticles with different concentrations.

Different optimization conditions and variables were used to optimize gold nanoparticle synthesis. HAuCl_4_.3H_2_O and *P. harmala* leaf extracts were combined at various ratios to examine Au nanoparticles. After that, these solutions were placed in various conditions for optimization. The ratios i.e., 2.1, 3.1, 4.1, 6.1, and 7.1 of these solutions were used in the experiment, and it took some time to see how the color changed when exposed to UV–visible spectroscopy. The change in color was noted in the image below. The prominent color was dark blue as shown in Figure 7.

#### 3.4.1 UV–Visible spectroscopy of gold nanoparticles

**FIGURE 8:**
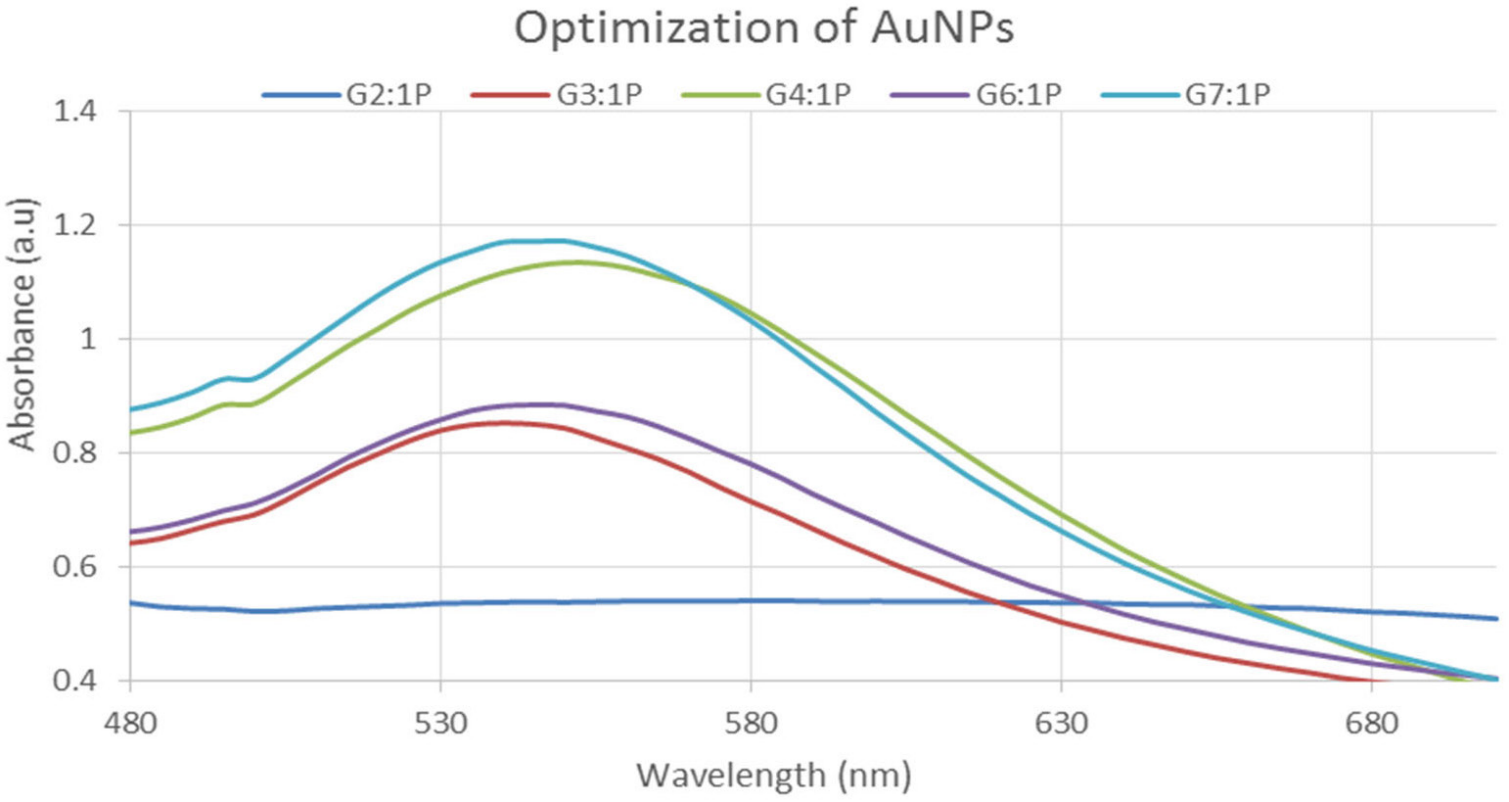
UV–Visible spectrum of gold nanoparticles synthesized by mixing leaves extracts.

Following the addition of *P. harmala* plant extract, the colorless gold chloride solution quickly turned to purple, indicating that a reaction had occurred and the gold ions had been converted to gold nanoparticles. This was only a visual cue concerning the creation of nanoparticles, which was later validated by UV–visible spectrophotometry. The maximum absorbance peak was seen at 535 nm for gold nanoparticles. Only the 4:1 ratio sample exhibited accurate stability results in all tests, according to the UV–visible spectrum. All of the peaks were virtually visible in the 500–600 nm range, as indicated in Figure 8, and aqueous solutions in different ratios.

#### 3.4.2 Salt effect on gold nanoparticles

**FIGURE 9:**
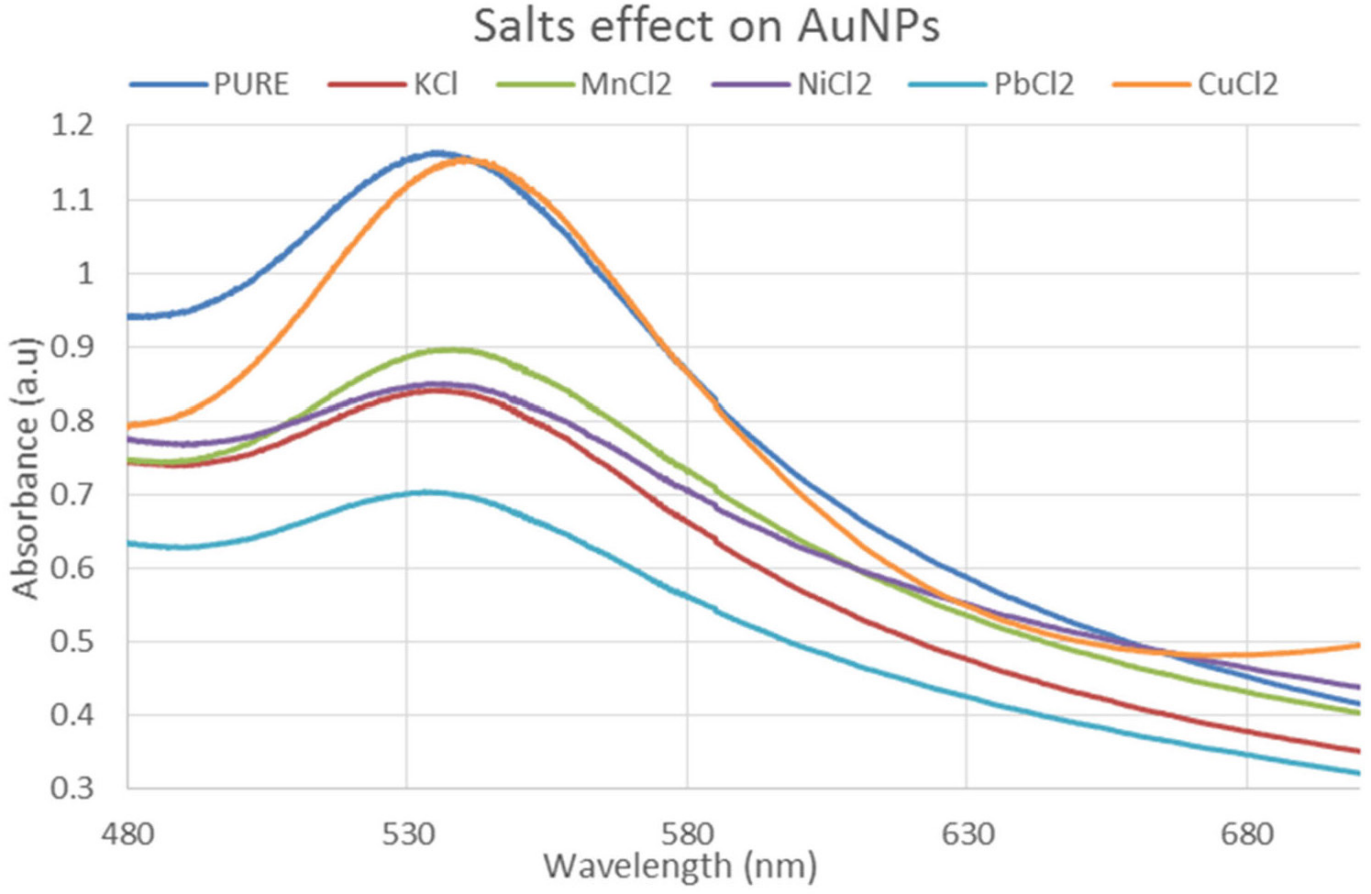
UV–Visible Spectrum revealing the effect of different salts on gold nanoparticles.

The impact of various ionic strengths on the size of gold NPs was also investigated. Because the important environmental circumstances may change depending on the salts and their ionic strength, examination of NPs in different salt ranges of ionic strength is also required for the assessment of NPs behavior in diverse salt environments. The salts used in the evaluation of the effects on gold NPs are NiCl_2_, CuCl_2_, KCl, MnCl_2_, and PbCl_2_. The differences in the particle size as a function of the ionic strength are shown in Figure 9. As can be seen from the graph's steep gradient, gold nanoparticles are more vulnerable to high salt strength than silver nanoparticles. The formation of aggregates in the medium is indicated by the size increase of nanoparticles.

#### 3.4.3 Heat effect on gold nanoparticles

**FIGURE 10:**
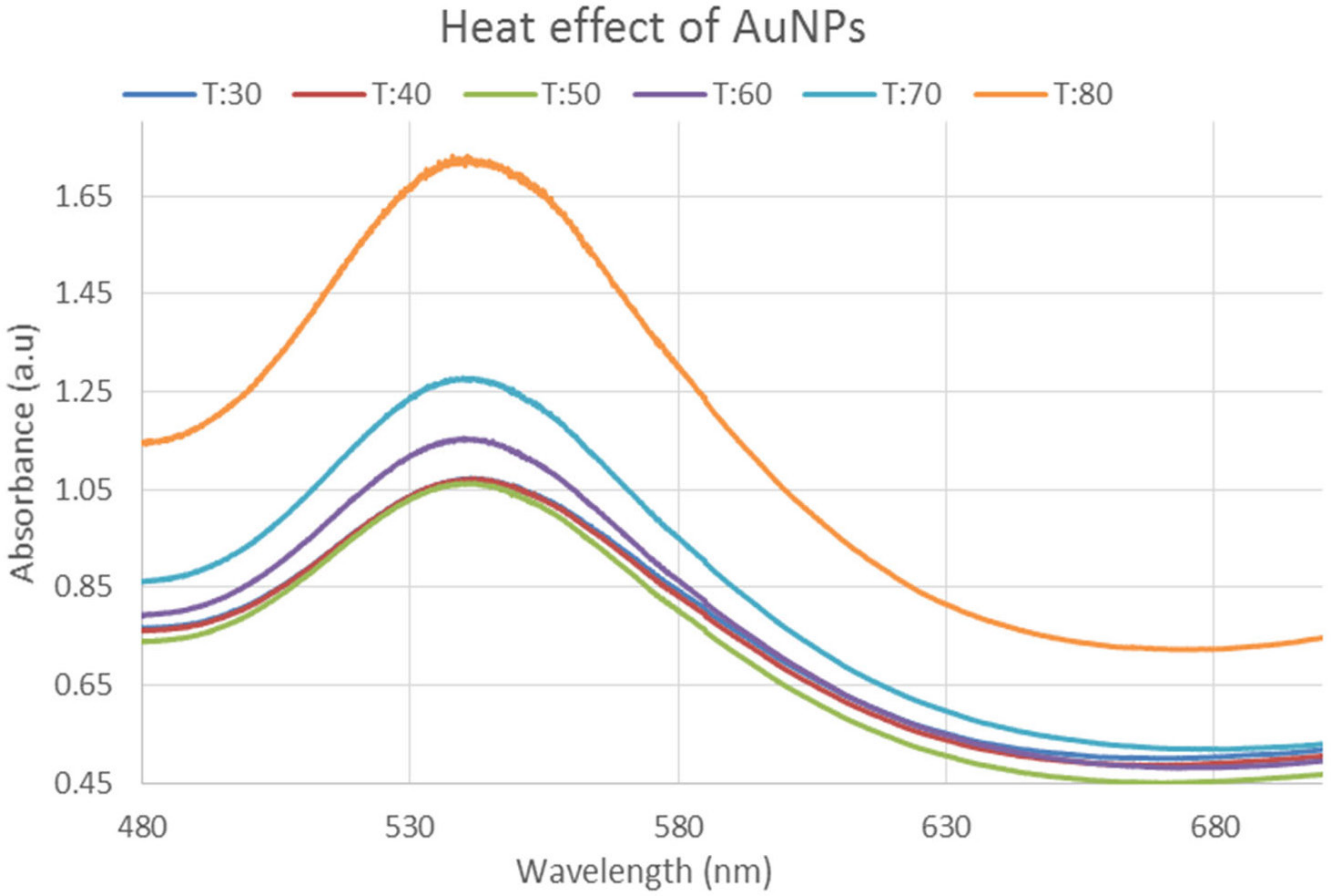
UV–Visible Spectra showing effect of heat at different temperatures on gold nanoparticles.

The gold nanoparticles were then heated at different temperatures to see what happens. The solution was exposed to a range of temperatures i.e., 30, 40, 50, 60, 70, and 80°C. Only two levels of temperature, 70 and 80°C, display a maximal absorbance peak of 1.65 and 1.25 a.u. at 560 nm wavelengths, as shown by the UV–visible spectroscopy results. The highest peaks are at 80°C, while the lowest peaks are at 30, 40, and 60°C, as illustrated in Figure 10. The findings reveal that the nanoparticles are more stable at temperatures between 30 and 80°C and that it is unstable at temperatures above 80°C due to dispersion as given in Figure 10.

#### 3.4.4 pH effect of gold nanoparticles

**FIGURE 11:**
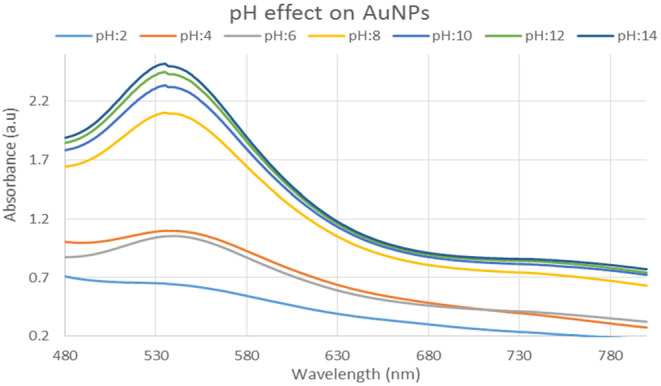
UV–Visible spectra showing the effect of different pH solution on gold nanoparticles.

This study shows that the production of gold nanoparticles is better suited to alkaline pH 8 and 12. The pH effect is shown in Figure 11.

#### 3.4.5 Effect of NaCl on gold nanoparticles

**FIGURE 12:**
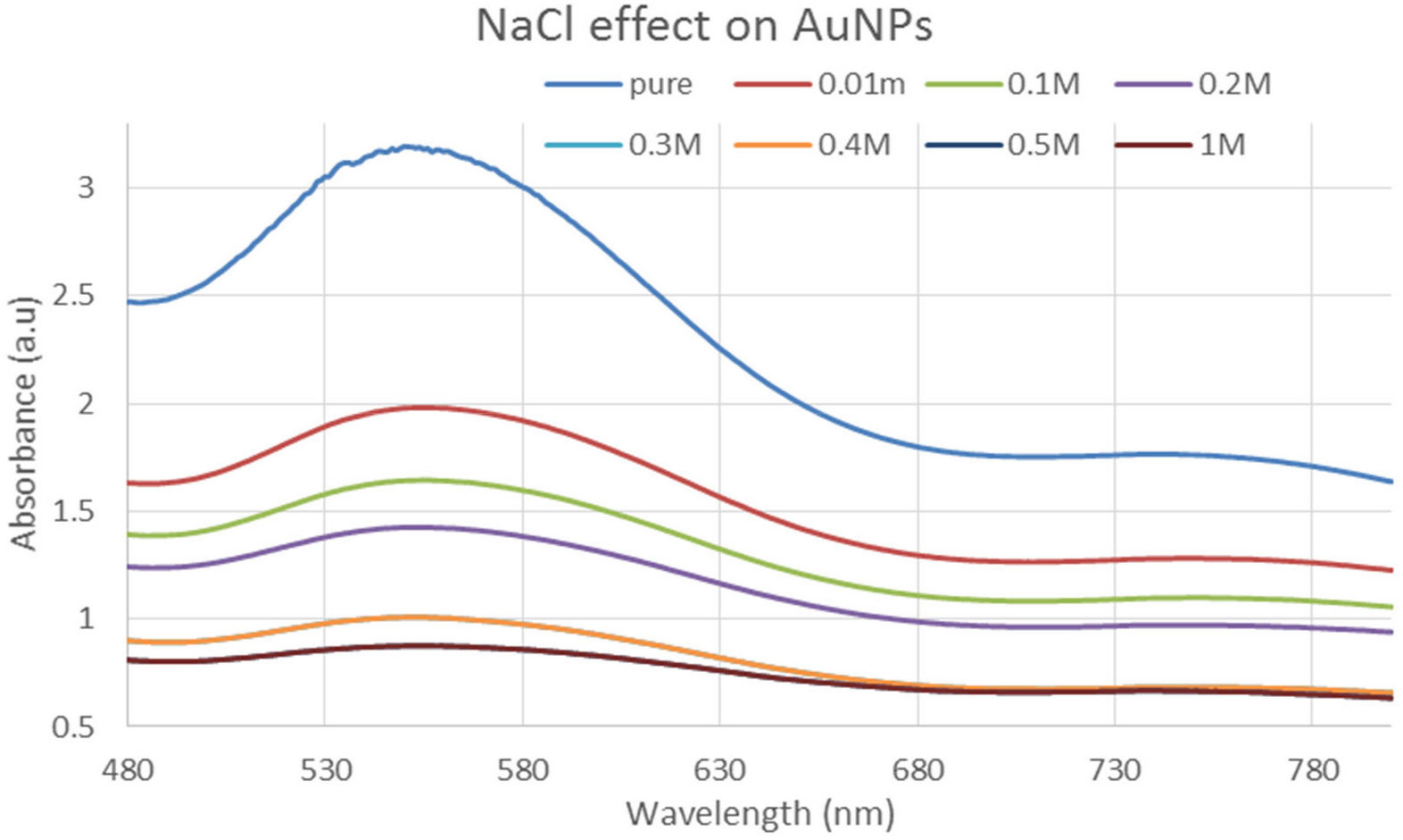
NaCl treatment with different concentrations in gold nanoparticle.

The gold nanoparticles made from leaf extracts were treated with varied amounts of sodium chloride (NaCl). UV–visible spectroscopy was used to investigate the effects of various solutions. Figure 12 depicts how the color of the sample changed at each concentration. For concentrations of 0.1, 0.5.1.0, 1.5, and 2.0, a UV–Visible spectrum was obtained. For all values between 2.25 and 2.3 a.u., the absorbance value is essentially identical. Furthermore, compared to the pure sample figure, all peaks for all samples show a decrease as shown in Figure 12.

### 3.5 Characterizations

#### 3.5.1 Fourier transform infrared (FTIR)

**FIGURE 13:**
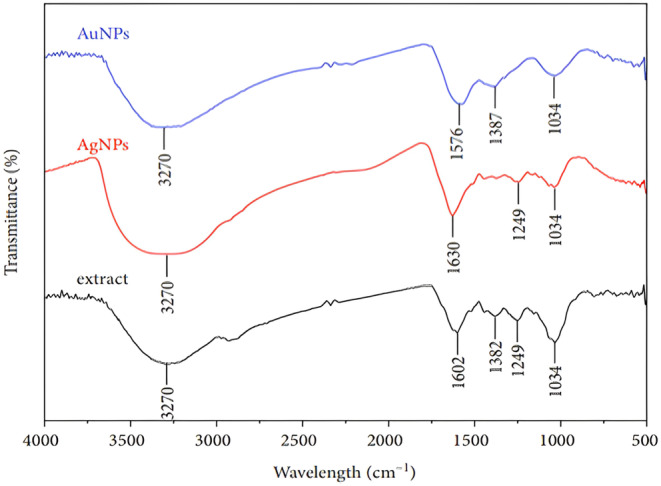
FTIR spectrum of *Peganum harmala* extracts, AgNPs and AuNPs.

The FTIR spectroscopy is commonly used for showing the presence of functional groups of bio‐molecules in the extract that behave as reducing and capping agents. Figure 13 reveals the FTIR spectra of the dried extract from the *P. harmala* plant and Ag and AuNPs. Accordingly, the FTIR spectrum of the dried extract and synthesized Ag and AuNPs appear nearly the same in the number of characteristic peaks, and their intensity for all samples was observed. The major bands of the *P. harmala* extract were recorded at 3270, 1602, 1382, 1249, and 1034 cm^−1^. The broad band at 3270 cm^−1^ is attributed to the stretching vibration of hydroxyl groups in polyphenolic compounds, terpenoids containing alcohol functional groups, and carboxylic compounds presented in the *P. harmala* leaves extract. The high‐intensity peak at 1602 cm^−1^ is related to C=C and C=O vibrations of benzene rings and carbonyl groups of amino acids. The peak at 1382 cm^−1^ is allocated to stretching vibrations of the carboxylate groups. The absorption band at 1249 cm^−1^ is related to hydroxyl groups in polyphenolic compounds with the in‐plane bending vibration. The peak at 1034 cm^−1^ is point out with the C–O band stretching of flavonoids. The small shift of several bands can be due to interactivity between functional groups of biomolecules and nanoparticles. It should be noted that the peak at 1382 cm^−1^in the FT‐IR spectrum of capped AgNPs was strongly reduced. Accordingly, respective polyphenolic groups are proven as the main reducing agents in the synthesis of MNP.

#### 3.5.2 SEM

**FIGURE 14:**
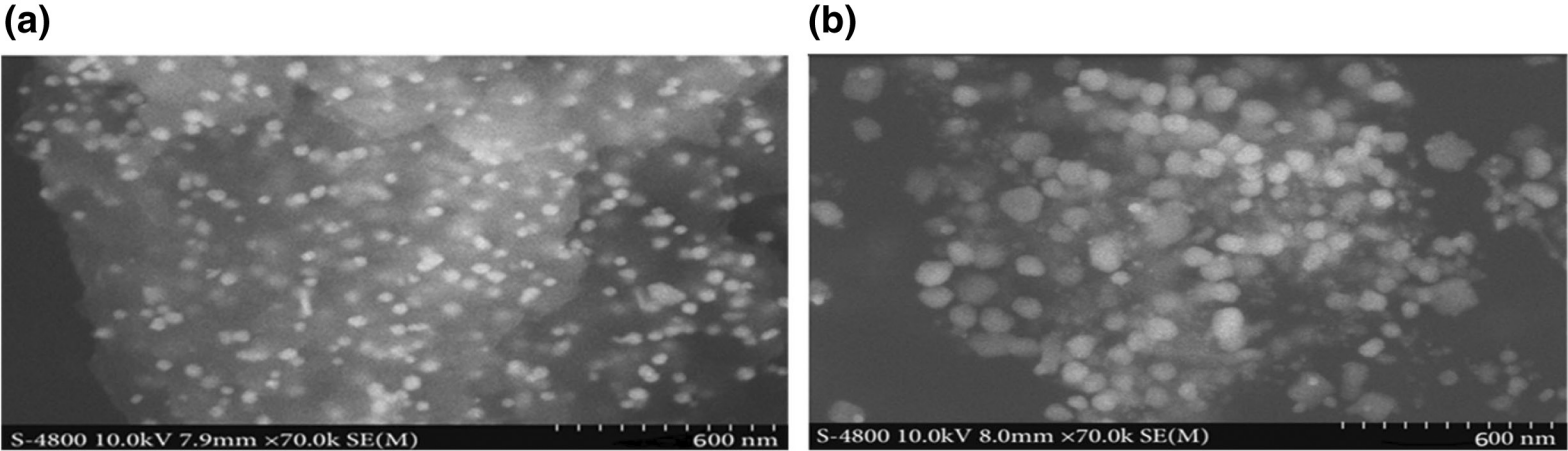
SEM image of Ag NPs (a), Au NPs (b) from *Peganum harmala* leaves extracts.

The *P. harmala* plant extracts synthesized silver nanoparticles by the green method were investigated by scanning electron microscopy to confirm the structure analysis. SEM images revealed that the structure of synthesized nanoparticles is oval, spherical, rod type and average size is from 42 to 72 nm. Similarly, the gold nanoparticles had oval and spherical forms between 12.6 and 35.7 nm, as revealed in Figure 14.

#### 3.5.3 Energy dispersive X‐ray spectroscopy of Ag and Au NPs

**FIGURE 15:**
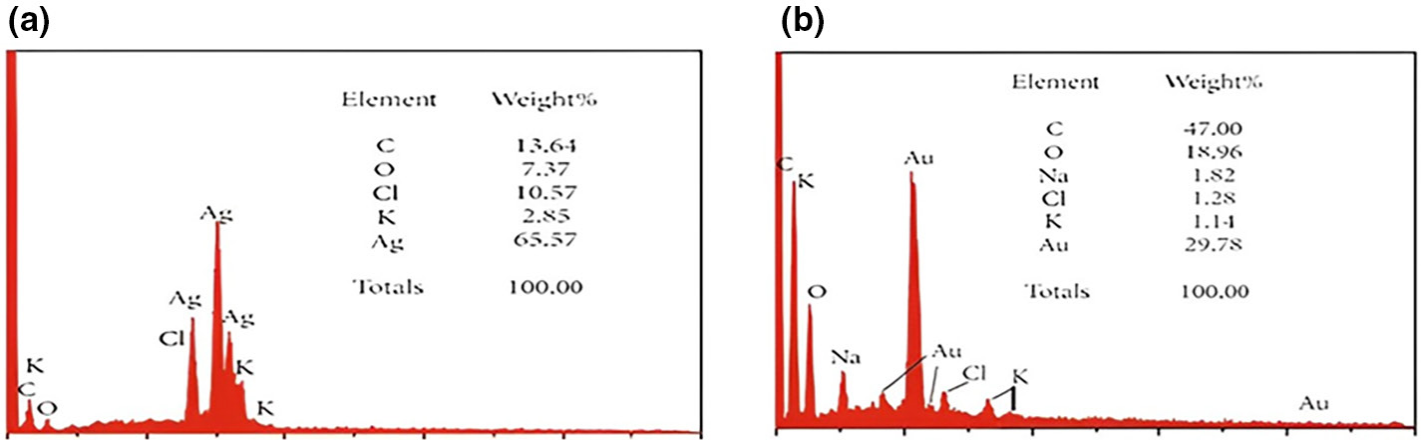
EDX of Ag (a), Au (b) NPs.

Energy dispersive X‐ray absorption spectrum of AgNPs synthesized from naturally reducing compounds present in aqueous leaves extract *P. harmala*. A large signal for Ag was discovered at 1.0 keV, as well as several weak signals for K, C, and Cl graphs. During chemical examination, the EDX for Au revealed the presence of various chemicals in leaf extract nanoparticles such as Zn, Cl, C, O, Au, k, and Au. As can be seen in the graph, C and Au received a strong signal at 1.0 keV, while Cl, K, O, and Na received faint signals. The EDX spectrum is provided in Figure 15.

### 3.6 Biological activities

#### 3.6.1 Anti‐bacterial

Antibacterial activities of *P. harmala* extract were studied, which produced inhibition zones of 19, 18, 20 , 21, and 17 mm against *Bacillus subtilis*, *S. aureus*, *E. coli*, *Pseudomonas aeroginosa*, and *Salmonella typhi*, respectively. The green synthesized AgNPs of this plant showed activities as; 23, 24, 22, 20, and 25 mm, against *B. subtilis*, *S. aureus*, *E. coli*, *P. aeroginosa*, and *S. typhi*, respectively. Gold nanoparticles of the same showed 20, 22, 23, 21, and 23 mm against the above‐mentioned bacterial strains, respectively (Table 2). The control (levofloxacin) showed a 100 mm zone of inhibition (Table 2). The plant extract of *P. harmala* showed good activities against selected bacterial strains compared to the standard control levofloxacin, but *S. typhi* was found resistant to plant extract of *P. harmala*.

**TABLE 2:** Antibacterial activity of *Peganum harmala* extract, its Silver and gold nanoparticles against selected bacterial strains.

S.NO	Samples	*Bacills subtilis*	*Staphylococcus aureus*	*Escherichia coli*	*Pseudomonas aeroginosa*	*Salmonella typhi*
		Zone of inhibition (mm)				
1	Plant extract of *P. harmala*	19	18	20	21	17
2	AgNPs of *P. harmala*	23	24	22	20	25
3	AuNPs of *P. harmala*	20	22	23	21	22
4	Standard control (Levofloxacin)	100	100	100	100	100

**FIGURE 16:**
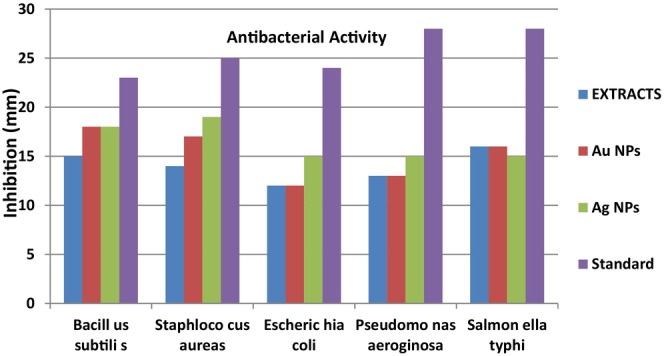
Anti‐bacterial activity of the synthesized AgNPs and AuNPs by using plant extracts.

The synthesized AgNPs showed good activities against selected bacterial strains compared to the standard control levofloxacin, but *P. aeroginosa* was found resistant to the synthesized AgNPs of *P. harmala*. Similarly, the synthesized AuNPs also showed good activities against selected bacterial strains compared to the standard control levofloxacin, however *B. subtilis* was found resistant to the synthesized AuNPs of *P. harmala* as shown in Table 2 and Figure 16.

#### 3.6.2 Anti‐fungal activity

**FIGURE 17:**
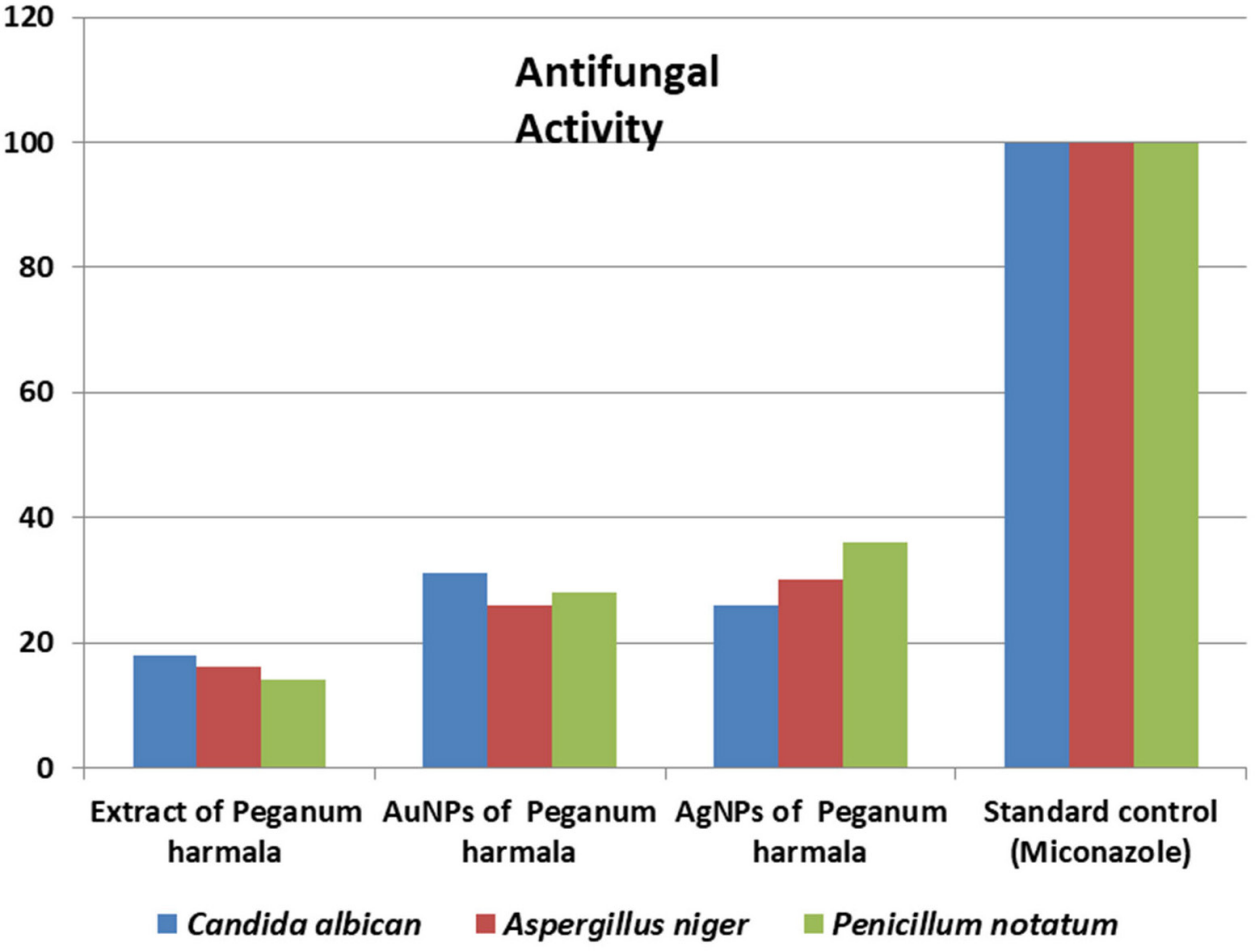
*Anti‐fungal* activity of the synthesized AgNPs and AuNPs by using plant extracts.

The anti‐fungal activity of silver and gold nanoparticles synthesized from *P. harmala* leaf extracts is investigated using a microdilution technique. *C. albicans* and *Aspergillus Niger* were among the fungus species used in the experiment. The anti‐fungal activity of *P. harmala* extracts and synthesized nanoparticles were investigated in the 43‐growing medium. Antifungal activity of synthesized nanoparticles and *P. harmala* extracts against *C. albicon* (18 mm), *A. Niger* (16 mm), and *Penicillium notatum* was discovered (14 mm). Synthesized gold nanoparticle of *P. harmala* leaves extracts demonstrate 31, 26, and 28 mm zone of inhibition. Similarly, silver nanoparticle shows 26, 30, and 36 mm. The antifungal activity results are shown in Table 3 and Figure 17.

**TABLE 3:** Anti‐fungal activity of the synthesized AgNPs and AuNPs by using plant extracts.

S.NO	Samples	*Candida albicans*	*Aspergillus niger*	*Penicillium notatum*
		Zone of inhibition (mm)		
1	Plant extract of *Peganum harmala*	18	16	14
2	AuNPs of *P. harmala*	31	26	28
3	AgNPs of *P. harmala*	26	30	36
4	Standard control (Miconazole)	100	100	100

## 4 DISCUSSION

Medicinal plants as a whole and their associated extracts from different parts are found to be significant in treating various metabolic syndromes. Plant extract‐based nanoparticles (NPs) are known as a significant approach for effective drug delivery (Abu‐Tahon et al., 2020; Aljabali et al., 2018; Iqbal et al., 2020). In modern medicine, the biosynthesis of plant‐based nanoparticles along with their promising bioactivities has gained significant interest of the scientific community. Plant‐extract‐based nanoparticles are preferred owing to their cost‐effectiveness and safety (Bawazeer et al., 2022). Literature shows that scientists have been biosynthesizing silver and gold nanoparticles from extracts of various plant parts like seeds, leaves, flower, bark, and roots (Kumar & Yadav, 2009). *P. harmala* extracts are found to be a rich source of phytochemicals that can be used for the treatment of various ailments. According to a study conducted by Qian et al. (2008), biosynthesized nanoparticles of *P. harmala* seeds extract acted as an alternative to traditional physical and chemical approaches owing to their eco‐friendly and economical effects. Moreover, Odeniyi et al. (2020) also synthesized nanoparticles using *P. harmala* plant extract and suggested a color change from yellow to red‐brown during the formulation of silver nanoparticles. Various studies have reported the synthesis of silver nanoparticles from extracts (methanolic and aqueous) of different parts of *P. harmala* including leaves, plants, and seeds (Alomar et al., 2020; Amin et al., 2014; Azizi et al., 2017).

In this study, varied ratios of the reaction mixture displayed various strengths of the absorption bands, demonstrating that the plant extract was used to create the silver nanoparticles. In the case of silver nanoparticles, a ratio 8:1 revealed maximum absorbance (425 nm) and indicated the presence of greater silver nanoparticles in the solution. Whereas, in the case of gold nanoparticles, sample ratio 4:1 showed stable results according to UV–Vis's spectra. Hence, the 8:1 (AgNPs) and 4:1 (AuNPs) ratio was further used for formulation of bulk solutions and further analysis. According to the UV–Vis spectrum, both the synthesized silver and gold nanoparticles were found to be more stable in CuCl_2_ as compared to other salt solutions. While, AgNPs and AuNPs were stable at 70 and 80°C, respectively. Moreover, both the AgNPs and AuNPs were most stable at pH 14. The reaction mixture's brightest color revealed the development of NPs and the absence of any reaction at low pH (Sathishkumar et al., 2010). The widening of the peaks was created at low pH ranges, which denotes the development of large‐sized NPs. Alkaline pH displays the narrowing of the peak at 400 nm with maximal sharp peak formation in the extract‐mediated synthesis of NPs. The development of the sharp peak is a sign that the nanoparticles' spherical shape has formed (Kumari et al., 2015). According to several studies, pH has a significant impact on how nanoparticle size and shape are controlled during synthesis.

The major bands of the *P. harmala* extract were recorded at 3270, 1602, 1382, 1249, and 1034 cm^−1^. The broad band at 3270 cm^−1^ is attributed to the stretching vibration of hydroxyl groups in polyphenolic compounds, terpenoids containing alcohol functional groups, and carboxylic compounds presented in the *P. harmala* leaves extract. The high‐intensity peak at 1602 cm^−1^ is related to C=C and C=O vibrations of benzene rings and carbonyl groups of amino acids (Motzer et al., 2018). The peak at 1382 cm^−1^ is allocated to stretching vibrations of the carboxylate groups. The absorption band at 1249 cm^−1^ is related to hydroxyl groups in polyphenolic compounds with the in‐plane bending vibration (Odeniyi et al., 2020). The peak at 1034 cm^−1^ is points out with the C–O band stretching of flavonoids (Gopinath et al., 2020).

Synthesized nanoparticles are oval, spherical, and rod‐type and average size is from 42 to 72 nm. Similarly, the gold nanoparticles had oval and spherical forms between 12.6 and 35.7 nm. The results of our study were similar to those reported in the literature (Yeamin, 2012). The formation of aggregates in the medium is indicated by the increase in the size of nanoparticles. The spectra's steepness depicts particle aggregation produced by ionic strength. The impacts of various electrolytes on nanoparticles were investigated and compared; concluding that salts containing divalent cations have a greater impact on NP aggregation than salts containing monovalent cations (Baalousha et al., 2013). The aggregation of NPs was also aided by the chloride ions. The aggregation of NPs demonstrated that divalent electrolytes effectively destabilized NPs (Huynh & Chen, 2011). The graph's steepness depicts particle aggregation produced by ionic strength (Baalousha et al., 2013). Nanoparticles investigated and compared the impacts of various electrolytes, concluding that salts containing divalent cations have a greater impact on NP aggregation than salts containing monovalent cations. The aggregation of NPs was also aided by the chloride ions. Likewise, Badawy also looked at gold nanoparticles and the effects of various monovalent and divalent cations (Badawy et al., 2010).

The green synthesized AgNPs of this plant showed activities as; 23, 24, 22, 20, and 25 mm, against *B. subtilis*, *S. aureus*, *E. coli*, *P. aeroginosa*, and *S. typhi*, respectively. Gold nanoparticles of the same showed 20, 22, 23, 21, and 23 mm against the above‐mentioned bacterial strains, respectively. Synthesized gold nanoparticle of *P. harmala* leaves extracts demonstrate 31, 26, and 28 mm zone of inhibition. Similarly, silver nanoparticle shows 26, 30, and 36 mm. Formulation of gold and silver NPs is currently being used to synthesize plant‐based NPs having boosted medicinal activities (Sadowski, 2010). The results of our studies are in agreement with previous findings of Bawazeer et al. (2022). They assessed the antibacterial and antifungal properties of black pepper‐based nanoparticles. According to them, black pepper‐based nanoparticles possessed significant effects against four different strains (Bawazeer et al., 2022).

## 5 CONCLUSIONS

The research work can be concluded as the best‐optimized ratios for AgNPs and AuNPs which were 7:1 and 4:1 respectively. Scanning electron microscopy confirmed the synthesis of AgNPs and AuNPs, and EDX confirmed its elemental presence. The AgNPs and AuNPs at 70 and 80°C showed intense peaks. All salts used e.g. MnCl_2_, KCl, PbCl_2_, NiCl_2_, CuCl_2_, and NaCl decreased the intensity of AgNPs and AuNPs peaks. The AgNPs had maximum absorbance at pH: 14 and that of AuNPs at pH: 14. The NPs showed enhanced activities against different fungi and bacteria than plant extract.

## AUTHOR CONTRIBUTIONS

**Izaz Ullah:** Conceptualization (equal); data curation (equal); formal analysis (equal); methodology (equal); validation (equal); writing – original draft (equal). **Abdur Rauf:** Conceptualization (equal); data curation (equal); formal analysis (equal); project administration (equal); supervision (equal); validation (equal); visualization (equal); writing – review and editing (equal). **Anees Ahmed Khalil:** Conceptualization (equal); data curation (equal); formal analysis (equal); investigation (equal); supervision (equal); validation (equal); visualization (equal); writing – original draft (equal); writing – review and editing (equal). **Muhammad Luqman:** Conceptualization (equal); methodology (equal); validation (equal); visualization (equal); writing – original draft (equal). **Md. Rezaul Islam:** Conceptualization (equal); data curation (equal); formal analysis (equal); methodology (equal). **Hassan A. Hemeg:** Conceptualization (supporting); data curation (supporting); investigation (supporting); validation (equal); visualization (equal); writing – original draft (equal). **Zubair Ahmad:** Conceptualization (equal); data curation (equal); formal analysis (equal); methodology (equal). **Yahya S. Al‐Awthan:** Conceptualization (equal); data curation (supporting); formal analysis (supporting); project administration (supporting); validation (equal); visualization (equal). **Omar Bahattab:** Conceptualization (supporting); data curation (equal); formal analysis (supporting); investigation (equal); writing – original draft (equal). **Mohammed Mansour Quradha:** Conceptualization (equal); data curation (equal); investigation (equal); writing – original draft (equal).

## CONFLICT OF INTEREST STATEMENT

All the authors declare no conflict of interest.

## Data Availability

The data that support the findings of this study are available on request from the corresponding author.
